# Anatomic fat depots and cardiovascular risk: a focus on the leg fat using nationwide surveys (KNHANES 2008–2011)

**DOI:** 10.1186/s12933-017-0536-4

**Published:** 2017-04-26

**Authors:** Eugene Han, Yong-ho Lee, Byung-Wan Lee, Eun Seok Kang, In-Kyu Lee, Bong-Soo Cha

**Affiliations:** 1 Division of Endocrinology, Department of Internal Medicine, Yonsei University College of Medicine, 50-1 Yonsei-ro, Seodaemun-gu, Seoul, 03722 South Korea; 2 Graduate School, Yonsei University College of Medicine, Seoul, South Korea; 3 Institue of Endocrine Research, Yonsei University College of Medicine, Seoul, South Korea; 4 Division of Endocrinology, Department of Internal Medicine, Kyungpook National University School of Medicine, Daegu, South Korea; 5 Division of Endocrinology, Department of Internal Medicine, Keimyung University School of Medicine, Daegu, South Korea

**Keywords:** Cardiovascular disease risk factors, Risk score, Obesity, Metabolic syndrome

## Abstract

**Background:**

Although central fat is a well-known risk factor for cardiovascular disease (CVD) and cardiometabolic disorders, the effect of other regional fats or muscle distribution on CVD risk has not been fully investigated.

**Methods:**

This was a cross-sectional study using nationally representative samples of 15,686 subjects from the 2008–2011 Korea National Health and Nutrition Examination Survey. Individual CVD risk was evaluated in adults aged ≥20 without prior CVD, using atherosclerotic cardiovascular disease (ASCVD) risk equations according to the 2013 ACC/AHA guidelines. Body composition was assessed by dual X-ray absorptiometry.

**Results:**

Ratio of leg fat to total fat (LF/TF ratio) was the most predictive for CVD among body fat or muscle distribution parameters (AUC = 0.748, 95% CI 0.741–0.755). ASCVD risk score was gradually increased with decreased LF/TF ratio (P < 0.001), and individuals whose LF/TF ratio in lowest tertile tended to belong to the high-risk (10-year risk >10%) group compared to those in the highest tertile (OR = 6.25, 95% CI 5.60–6.98). Subjects in the lowest tertile showed increased risk of cardiometabolic risk factor components including obesity, hypertension, diabetes, dyslipidemia, chronic kidney disease, and albuminuria (OR range 2.57–11.24, all P < 0.001). In addition, a higher LF/TF ratio was associated with decreased ASCVD risk, even in subjects with multiple CVD risk factors. Multiple logistic regression analyses also demonstrated this association (OR = 1.85, 95% CI 1.36–2.52).

**Conclusions:**

Among various body composition parameters, LF/TF ratio was superior in predicting higher CVD risk and a higher LF/TF ratio was independently associated with decreased risk of CVD and each cardiometabolic risk factor.

**Electronic supplementary material:**

The online version of this article (doi:10.1186/s12933-017-0536-4) contains supplementary material, which is available to authorized users.

## Background

As obesity has become an increasingly epidemic disease, the incidence of cardiovascular and metabolic diseases have risen to become public health issues. Overweight and obesity are related to cardiovascular, and all-cause mortality [1]. More specifically, it has been demonstrated that excessive visceral fat is related to an increased risk of hypertension [2] and diabetes [3]. Although the association between excessive abdominal fat and risk of cardiovascular disease (CVD) and other cardiometabolic diseases has been established, investigation of a relationship between other body fat accumulation and cardiometabolic disease is limited. In addition, the different effect of regional body fat or muscle composition on CVD has not been fully understood.

Visceral adiposity is linked to altered myocardial glucose uptake [4], concentric left ventricular remodeling [5], and left atrial dysfunction [6]. In contrast, subcutaneous fat depot was associated with higher cardiac output and lower systemic vascular resistance [5]. Among subcutaneous fat, thigh girth or thigh fat is reported to attenuate risk for dyslipidemia [7] and glucose intolerance [8]. The protective effect of larger hip circumference to CVD has also been confirmed in previous studies of myocardial infarction [9], coronary heart disease (CHD) [10], and any CVD event [11]. Studies on lower-body fat have focused on the hip or thigh and indirectly assessed fat distribution by circumference. In addition, studies investigating on the effect of various body composition parameters on CVD risk are limited.

Dual-energy X-ray absorptiometry (DXA) has been recently used to assess body fat distribution as well as bone density. The regional fat measurement using DXA is reliable compared to computed tomography, a gold standard tool for measurement [12]. It also has the capability to scan whole body fat distribution with minimal radiation exposure.

We hypothesized that higher ratio of leg fat to total fat (LF/TF ratio) is related to low CVD risk regardless of other metabolic conditions. The aim of this study was to investigate the effect of each body fat or muscle distribution on CVD risk, and the association between LF/TF ratio and various CVD and cardiometabolic risk factor components in the general population.

## Methods

### Study population

**Fig. 1:**
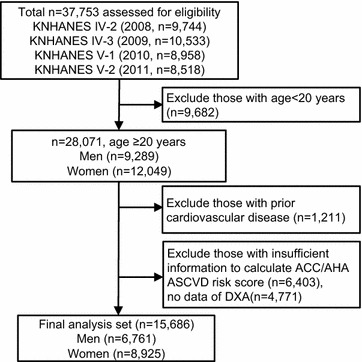
The flow diagram of subject inclusion and exclusion in the Korean National Health and Nutrition Examination Surveys (KNHANES IV and V)

This cross-sectional study utilized data collected from participants in the Korea National Health and Nutrition Examination Surveys (KNHANES) 2008–2011. This nationwide, population-based, annual survey is conducted by the Korean government to monitor public health [13]. In KNHANES data, medical history includes smoking habits, alcohol consumption, exercise level, and disease diagnosis and/or treatment, based on direct interviews and self-reporting. Each KNHANES is comprised of independent data sets from the general population. As described in Fig. 1, among 37,753 participants, we initially selected those aged ≥20 without a prior history of CVD. We excluded those with missing data for DXA and CVD risk assessments, such that, 15,686 subjects (6761 men and 8925 women) were included in the analysis. All participants provided written informed consent to participate in the original KNHANES. The survey protocol was approved by the institutional review board of the Korean Centers for Disease Control and Prevention (2008-04EXP-01-C, 2009-07CON-03-2C, 2010-02CON-21-C, and 2011-02CON-06C).

### Regional body distributions and cardiovascular disease risk

Fat mass and muscle mass were measured using DXA (QDR 4800A; Hologic Inc., Bedford, MA, USA). The DXA scans provided soft tissue and bone measurements for both arms and both legs, and the trunk. Values for these regions include total mass, fat mass, muscle mass. Leg measurement was assessed by sum of both legs including thigh and calf and arm measurement was indicated as sum of both side upper arms and forearms values. CVD risk was evaluated using various approaches, including the 10-year atherosclerotic cardiovascular disease (ASCVD) risk score from the 2013 American College of Cardiology/American Heart Association (ACC/AHA) guidelines [14]. In addition, the Framingham 10-year CVD risk score [15] and the Korean coronary heart disease (CHD) prediction model [16] were assessed. Leg muscle mass and each CVD risk score was analyzed and categorized into sex-specific tertiles. Moreover, LF/TF ratio was divided into tertiles according to obesity (body mass index [BMI] ≥25 kg/m^2^, Asian-pacific criteria [17]) and sex. Individuals exhibiting ACC/AHA ASCVD risk >10%, Framingham CVD risk >20%, and Korean CHD risk >5% were classified into the high-risk group. We assessed regional body fat and muscle distributions’ receiver operator characteristic (ROC) curve to predict and high CVD risk. In addition, calculations of the area under the ROC curve (AUC) were compared among the fat and muscle parameters.

### Cardiometabolic disease components

During surveys, blood pressure was manually measured using mercury sphygmomanometers (Baumanometer; W.A. Baum, Copiague, NY) three times in resting, seated positions, and final blood pressure values were assessed by averaging the second and third blood pressure readings. Overnight (≥8-h) fasting blood and spot urine samples were collected, refrigerated, and transported to a central laboratory (NeoDin Medical Institute, Seoul, South Korea) within 24 h. Diabetes was defined as using anti-diabetic agents, or who exhibited fasting plasma glucose ≥126 mg/dL or glycated hemoglobin 6.5%. Individuals were considered to have hypertension if their systolic and/or diastolic blood pressure was ≥140/90 mmHg, or if they were taking anti-hypertensive medications. Central obesity was defined as waist circumference ≥90 cm in men and ≥85 cm in women [18]. Metabolic syndrome was based on the modified National Cholesterol Education Program Adult Treatment Panel III criteria, adopted for the Korean population [19]. Hyper low density lipoprotein (LDL) cholesterolemia was characterized as LDL ≥160 mg/dL for both sexes, and hypo high density lipoprotein (HDL) cholesterolemia was defined as HDL <40 mg/dL for men, and <50 mg/dL for women. Hypertriglyceridemia was defined as serum triglycerides ≥150 mg/dL or taking triglyceride lowering agents. Estimated glomerular filtration rate (eGFR) was calculated using the Chronic Kidney Disease Epidemiology collaboration equation [20], and chronic kidney disease was characterized as subjects with eGFR <60 mL/min/1.73 m^2^ [21]. Albuminuria was characterized as having more than one positive dip-stick test or urine albumin-to-creatinine ratio >30 mg/g [21]. Excluding individuals with missing urine data, 819 subjects were categorized into sex-specific LF/TF ratio tertiles and analyzed for risk of albuminuria. Regular exercise was defined as “engaging in intense physical activity, that made one very tired or breathe much harder than usual, and involved more than 20 min per session at least 3 times per week” (e.g., running, jogging, mountain climbing, fast cycling, fast swimming, playing soccer, playing basketball, playing squash or singles tennis, transporting heavy objects, etc.). An age cutoff point to classify adults as younger or older was 50 years, the median value.

### Statistical analysis

Data are presented as the mean ± standard deviation for continuous variables, and as number, or percent for categorical variables. Simple correlation coefficients were used to assess correlations among regional fat depots. We analyzed participants’ characteristics according to LF/TF ratio tertiles, using one-way analysis of variance (ANOVA) to compare continuous variables and, χ^2^ tests for categorical variables, followed by post hoc analyses using the Bonferroni method. To evaluate the association between LF/TF ratio and CVD risk, we attempted to minimize the effects of comorbidities. Subjects were classified according to the presence of hypertension, diabetes, metabolic syndrome, and obesity, and then χ^2^ tests were applied to each group. Multivariable adjusted logistic regression analyses were used to test the independent association between LF/TF ratio and high CVD risk (ACC/AHA ASCVD risk >10%, Framingham CVD risk >20% or Korean CHD risk >5%) and between LF/TF ratio and CVD risk factors. To measure the prediction accuracy of each body compositions for high CVD risk, we calculated and compared AUC of each regional fat parameters. The comparison of the AUC for ROC curves was performed on the basis of the Delong test [22]. As total cholesterol, triglyceride, HDL cholesterol, LDL cholesterol, insulin, and the homeostasis model assessment of insulin resistance (HOMA-IR) values were not normally distributed, analyses were performed using log-transformed data in order to achieve approximately symmetrical distributions. Statistical analyses were performed using IBM SPSS version 23.0 for Windows (IBM Corp., Armonk, NY, USA), and MedCalc (version 13.1; http://medcalc.software.informer.com/13.1); P < 0.05 was considered statistically significant.

## Results

### Baseline characteristics by ratio of leg fat to total fat

A total of 15,686 individuals with no history of CVD were analyzed in the present study (Fig. 1). Individuals with a higher LF/TF ratio tended to have smaller waist circumference, lower blood pressure, and more favorable metabolic conditions (glucose, insulin, HOMA-IR, LDL cholesterol, triglyceride, HDL cholesterol, and eGFR) (Table 1). Subsequently, a low prevalence of hypertension, diabetes, metabolic syndrome and chronic kidney disease was observed in the highest LF/TF ratio tertile in both non-obese and obese population. The correlation between leg fat and arm fat was strong (Pearson coefficient = 0.808, P < 0.001), while the association between leg fat and trunk fat was relatively weak (Pearson coefficient = 0.647, P < 0.001) in our population.

**Table 1:** Baseline characteristics of study population by categories of ratio of leg fat to total fat (LF/TF ratio)

	Tertiles of LF/TF ratio					
	Lean			Obese		
	Lowest tertile (n = 3592)	Second tertile (n = 3590)	Highest tertile (n = 3593)	Lowest tertile (n = 1631)	Second tertile (n = 1627)	Highest tertile (n = 1633)
Leg fat (kg)	4.3 ± 1.4	5.1 ± 1.7^§^	5.4 ± 1.8^§,∥^	5.1 ± 1.4	6.4 ± 1.7^§^	7.6 ± 2.1^§,∥^
Total fat (kg)	15.9 ± 4.1	15.6 ± 4.4^§^	13.9 ± 4.0^§,∥^	21.5 ± 4.9	22.6 ± 5.3^§^	23.0 ± 5.4^§^
LF/TF ratio (%)	26.4 ± 3.2	32.5 ± 3.3^§^	38.7 ± 4.8^§,∥^	23.8 ± 2.2	28.1 ± 1.8^§^	33.0 ± 3.2^§,∥^
Age (year)	57.0 ± 13.5	47.5 ± 15.0^§^	38.2 ± 14.2^§,∥^	55.7 ± 12.3	51.1 ± 13.9^§^	43.6 ± 14.6^§,∥^
Men (%)	40.4	40.3	40.3	49.2	49.2	49.2
Waist circumference (cm)	80.4 ± 6.5	76.7 ± 6.9^§^	72.5 ± 7.1^§,∥^	92.0 ± 6.8	91.0 ± 7.0^§^	88.6 ± 7.3^§,∥^
BMI (kg/m^2^)	22.7 ± 1.7	22.0 ± 1.9^§^	20.9 ± 2.1^§,∥^	27.4 ± 2.1	27.5 ± 2.3	27.2 ± 2.2^∥^
Systolic blood pressure (mmHg)	122.1 ± 17.9	114.0 ± 16.6^§^	107.8 ± 13.6^§,∥^	125.9 ± 16.3	122.8 ± 16.4^§^	117.4 ± 15.4^§,∥^
Diastolic blood pressure (mmHg)	76.2 ± 10.4	73.1 ± 10.2^§^	70.1 ± 9.3^§,∥^	79.9 ± 10.2	78.9 ± 10.4^§^	76.5 ± 10.2^§,∥^
Fasting plasma glucose (mg/dL)	102.1 ± 26.6	93.6 ± 19.3^§^	89.3 ± 12.2^§,∥^	111.0 ± 31.8	101.6 ± 21.0^§^	95.2 ± 15.9^§,∥^
Total cholesterol (mg/dL)^a^	194.4 ± 36.6	185.2 ± 33.2^§^	172.9 ± 30.0^§,∥^	200.3 ± 37.4	198.3 ± 36.5	191.3 ± 35.3^§,∥^
HDL cholesterol (mg/dL)^a^	50.7 ± 12.0	54.6 ± 13.0^§^	57.5 ± 12.6^§,∥^	47.3 ± 10.8	47.7 ± 11.1	49.8 ± 11.0^§,∥^
Triglycerides (mg/dL)^a^	154.4 ± 114.1	115.6 ± 87.4^§^	85.1 ± 67.8^§,∥^	190.6 ± 150.1	174.0 ± 121.3^§^	136.3 ± 98.3^§,∥^
LDL cholesterol (mg/dL)^a^	118.4 ± 33.5	112.5 ± 29.5^§^	102.8 ± 26.6^§,∥^	122.1 ± 34.0	122.0 ± 32.5	119.6 ± 31.3
eGFR (mL/min/1.73 m^2^)	90.1 ± 16.1	97.3 ± 16.5^§^	103.6 ± 16.1^§,∥^	98.1 ± 16.8	92.2 ± 16.2^§^	97.9 ± 16.6^§,∥^
WBC (10^3^/μL)	6.1 ± 1.7	5.8 ± 1.6^§^	5.6 ± 1.5^§,∥^	6.6 ± 1.8	6.5 ± 1.7	6.2 ± 1.6^§,∥^
Insulin (ųU/mL)^a^	9.4 ± 5.4	8.7 ± 3.9^§^	8.4 ± 3.4^§,∥^	13.0 ± 7.6	12.1 ± 5.8	11.1 ± 4.6
HOMA-IR^a^	2.4 ± 2.3	2.0 ± 1.1^§^	1.9 ± 0.8^§,∥^	3.7 ± 3.4	3.1 ± 1.9^§^	2.6 ± 1.2^§,∥^
Alcohol (g/week)	73.8 ± 122.5	63.5 ± 113.3^§^	44.6 ± 84.2^§,∥^	82.0 ± 132.0	78.9 ± 123.5	67.8 ± 113.4^§^
Current smoker (%)	20.5	21.6	20.9	21.3	23.3	24.2
Regular exercise (%)^b^	24.2	24.7	22.0^§^	26.4	27.2	28.5
Hypertension (%)	37.5	17.9^§^	7.0^§,∥^	53.8	40.7^§^	24.0^§,∥^
Diabetes (%)	15.2	4.1^§^	1.1^§,∥^	26.4	12.2^§^	4.3^§,∥^
Metabolic syndrome (%)	37.6	11.9^§^	2.4^§,∥^	72.4	60.2^§^	35.0^§,∥^
Chronic kidney disease (%)^c^	4.3	2.1^§^	0.6^§,∥^	5.3	3.7	2.2^§^

Data for continuous variables were expressed as mean ± standard deviation, and categorical variables were expressed as percent (%)

*BMI* body mass index; *HDL* cholesterol, high density lipoprotein cholesterol; *LDL* cholesterol, low density lipoprotein cholesterol; *eGFR* estimated glomerular filtration rate; *WBC* white blood cell; *HOMA-IR* homeostasis model assessment of insulin resistance

^§^P < 0.05 by post hoc analyses when compared with lowest tertile

^∥^P < 0.05 by post hoc analyses when compared with second tertile

^a^ Log-transformed

^b^ Regular exercise was defined as engaging in intense physical activity, that made one very tired or breathe much harder than usual, and involved more than 20 min per session at least 3 times per week

^c^ Chronic kidney disease was defined if subjects had an eGFR less than 60 ml/min/1.73 m^2^

### Various fat depots and cardiovascular disease risk

**Fig. 2:**
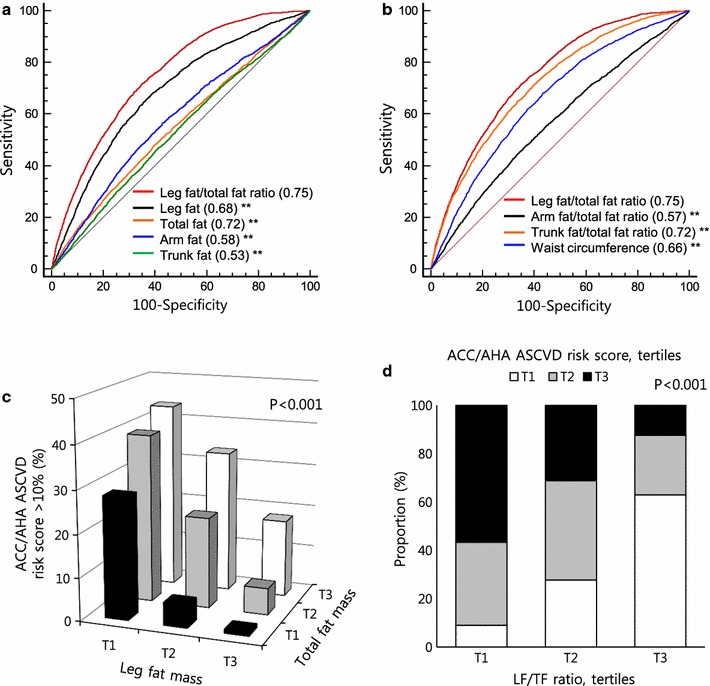
Regional body fat distributions, receiver operating characteristic (ROC) curves, and high ACC/AHA ASCVD risk. **a** ROC curves to predict high ACC/AHA ASCVD risk (>10%) for each regional fat. **b** ROC curves to predict high ACC/AHA ASCVD risk for other regional fat ratios and waist circumference. **c** Proportion of individuals with high ACC/AHA ASCVD risk by leg fat and total fat tertiles. **d** Proportion of individuals with ACC/AHA ASCVD risk score tertiles by LF/TF ratio. **P < 0.001

Leg fat was superior to arm, total, or trunk fat distributions in predicting higher values of ACC/AHA ASCVD risk scores (Fig. 2a). In addition, the LF/TF ratio (AUC = 0.748, 95% CI 0.741–0.755) showed greater accuracy in predicting high ACC/AHA ASCVD risk compared to other fat distributions (AUC_arm fat/total fat ratio_ = 0.568, 95% CI 0.561–0.576, AUC_trunk fat/total fat ratio_ = 0.717, 95% CI 0.709–0.724, AUC_waist circumference_ = 0.659, 95% CI 0.652–0.667) (Fig. 2b). After stratifying into sex-specific leg fat and total fat tertile separately, individuals with a higher leg fat even in higher total fat had shown lower ACC/AHA ASCVD risk compared to lower leg fat and lower total fat groups (Fig. 2c). LF/TF ratio tertiles were well-correlated with ACC/AHA ASCVD score tertiles (Fig. 2d). The other risk scores showed similar results (Additional file 1: Figure S1).

### Ratio of leg fat to total fat and cardiometabolic disease risk components

We have demonstrated a relationship between LF/TF ratio and CVD risk; likewise, we analyzed the risks of each cardiometabolic factor component as related to LF/TF ratio. In Table 2, individuals with the lowest LF/TF ratio tertile had the highest risk of obesity, hypertension, diabetes, dyslipidemia, metabolic syndrome, and kidney dysfunction (OR range 2.64–11.24, all P < 0.001). To assess the association between LF/TF ratio and CVD risk independent of other covariates, we performed multiple logistic regression analysis. After sequential adjustment for confounding covariates such as age, sex, obesity, smoking, alcohol, systolic blood pressure, serum glucose, dyslipidemia, BMI, menopause state and kidney function, individuals with lower LF/TF ratios were found to have significantly higher adjusted odds ratios (AORs) for high ACC/AHA ASCVD risk (AOR = 1.85, 95% CI 1.36–2.52, P < 0.001) compared to subjects with the highest LF/TF ratio. The AOR for high risk of CVD gradually increased in lower LF/TF ratio, in other models.

**Table 2:** Odds ratio and 95% confidential interval of cardiometabolic disease risk factors and high CVD risk according to sex-specific LF/TF ratio tertiles in adults

	LF/TF ratio		
	Highest tertile	Second tertile	Lowest tertile
Risk factor			
Obesity^a^	1 (referent)	3.72 (3.33–4.15)	7.67 (6.81–8.23)
Central obesity^b^	1 (referent)	4.29 (3.83–4.82)	9.22 (8.16–10.42)
Hypertension	1 (referent)	2.05 (1.80–2.34)	3.57 (3.14–4.07)
Diabetes	1 (referent)	2.88 (2.19–3.78)	8.89 (6.87–11.50)
Hyper LDL-cholesterolemia^c^	1 (referent)	2.14 (1.76–2.60)	2.72 (2.23–3.32)
Hypo HDL-cholesterolemia^d^	1 (referent)	1.92 (1.76–2.11)	2.91 (2.64–3.22)
Hypertriglyceridemia^e^	1 (referent)	3.01 (2.69–3.37)	6.14 (5.46–6.90)
Metabolic syndrome	1 (referent)	4.06 (3.53–4.67)	11.24 (9.78–12.93)
Chronic kidney disease^f^	1 (referent)	1.97 (1.30–2.99)	2.57 (1.73–3.81)
Albuminuria^g^	1 (referent)	2.03 (1.21–3.41)	2.64 (1.57–4.44)
High ACC/AHA ASCVD risk			
Crude	1 (referent)	2.53 (2.25–2.84)	6.25 (5.60–6.98)
Model 1	1 (referent)	1.95 (1.56–2.44)	3.69 (2.98–4.57)
Model 2	1 (referent)	1.65 (1.30–2.08)	2.91 (2.31–3.66)
Model 3	1 (referent)	1.26 (0.92–1.71)	1.85 (1.36–2.52)
High Framingham CVD risk			
Crude	1 (referent)	2.48 (2.16–2.84)	6.32 (5.56–7.18)
Model 1	1 (referent)	1.96 (1.61–2.39)	4.09 (3.39–4.94)
Model 2	1 (referent)	1.70 (1.38–2.09)	3.21 (2.63–3.92)
Model 3	1 (referent)	1.43 (1.07–1.91)	2.37 (1.79–3.15)
High Korean CHD risk			
Crude	1 (referent)	3.43 (2.30–5.13)	7.98 (5.48–11.63)
Model 1	1 (referent)	2.47 (1.59–3.82)	4.08 (2.69–6.17)
Model 2	1 (referent)	2.39 (1.52–3.75)	3.64 (2.37–5.60)
Model 3	1 (referent)	1.94 (1.11–3.38)	2.57 (1.52–4.37)

Risk factors are adjusted for age, sex, exercise, smoking, and alcohol drinking

Model 1: adjusted for age, and sex

Model 2: adjusted for age, sex, exercise, smoking, alcohol drink, and BMI

Model 3: adjusted for age, sex, exercise, smoking, alcohol drink, BMI, systolic blood pressure, fasting blood glucose, hyper LDL-cholesterolemia, eGFR, and menopause

^a^ Obesity was defined BMI ≥25 kg/m^2^

^b^ Central obesity was defined waist circumference ≥90 cm in men, ≥85 cm in women

^c^ Hyper LDL-cholesterolemia was characterized as calculated LDL cholesterol ≥160 mg/dL

^d^ Hypo HDL-cholesterolemia was defined as serum HDL cholesterol <40 mg/dL for men, and <50 mg/dL for women

^e^ Hypertriglyceridemia was defined as serum triglycerides ≥150 mg/dL or taking triglyceride lowering agents

^f^ Chronic kidney disease was defined if subjects had an eGFR less than 60 mL/min/1.73 m^2^

^g^ Albuminuria was characterized urine albumin-creatinine ratio >30 mg/g or urine protein ≥2 positive. The LF/TF ratio tertile was newly categorized in 819 subjects with urine test results

### Ratio of leg fat to total fat is associated with cardiovascular disease risks independent of hypertension, diabetes, and metabolic syndrome

The increasing average ACC/AHA ASCVD risk scores was linked with decreased LF/TF ratio in both non-obese and obese group, and this finding was shown in both young and old age group (Additional file 2: Figure S2). The distribution of high-risk subjects according to LF/TF ratio tertiles showed the same trend as ACC/AHA ASCVD risk scores regardless of age group (9.1, 20.1, and 38.4% for highest, second, and lowest tertiles, respectively, P < 0.001).

**Fig. 3:**
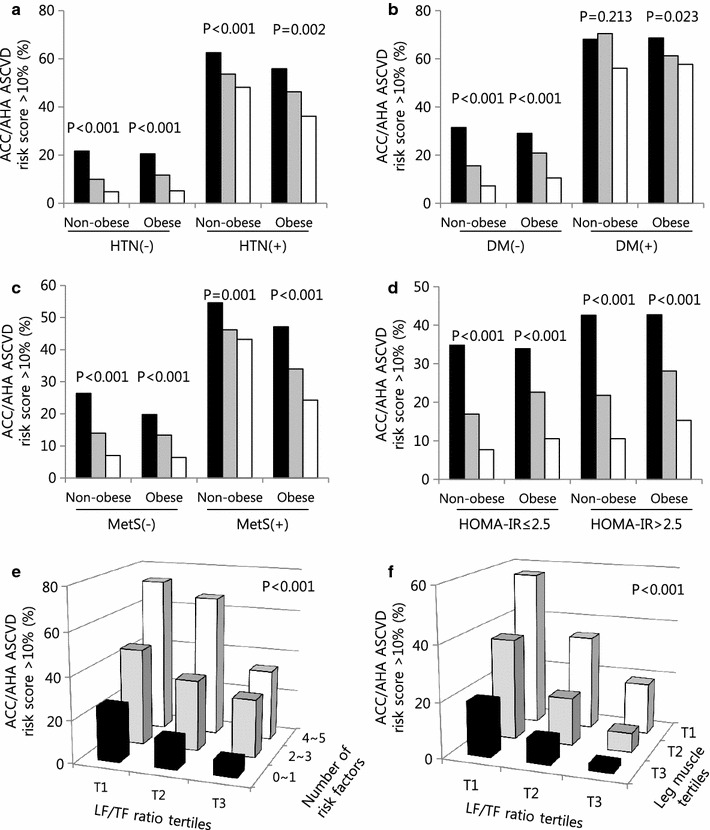
Difference in ACC/AHA ASCVD risk according to LF/TF ratio tertiles, subgroup analysis. Proportion of individuals with high ACC/AHA ASCVD risk (>10%) stratified by **a** hypertension, **b** diabetes, **c** metabolic syndrome, **d** insulin resistance (HOMA-IR), and **e** central obesity. **f** Number of cardiovascular risk factors according to LF/TF tertiles. Risk factors are obesity, hypertension, diabetes, hyper LDL-cholesterolemia, and hypertriglyceridemia. *HTN* hypertension, *DM* diabetes mellitus, *MetS* metabolic syndrome, *HOMA-IR* homeostasis model assessment of insulin resistance

Subgroup analyses were conducted to identify the LF/TF ratio and CVD risk independent of other metabolic conditions. Figure 3 depicts that, regardless of the presence of hypertension, diabetes, metabolic syndrome or insulin resistance state, a higher LF/TF ratio was associated with lower ACC/AHA ASCVD risks in both non-obese and obese group. When other CVD risk assessments were applied, similar results were observed (Additional file 3: Figure S3, Additional file 4: Figure S4). Furthermore, people with a higher LF/TF ratio were less likely to have multiple CVD risk factors. In individuals with a higher LF/TF ratio having multiple risk factors, their CVD risk was not as high as those with lower LF/TF ratios, regardless of CVD risk models (Fig. 3e). We further assessed ACC/AHA ASCVD risk in leg muscle mass in relation to LF/TF ratio. Along with a higher LF/TF ratio, higher leg muscle mass was associated with lower ACC/AHA ASCVD risk and both LF/TF ratio and leg muscle mass synergistically decreased ACC/AHA ASCVD risk (Fig. 3f).

## Discussion

This large, nationally representative, population-based study demonstrated that LF/TF ratio was superior to other parameters in predicting high CVD risk, and that people with a higher LF/TF ratio tended to have decreased risk of CVD and other cardiometabolic risk components. LF/TF ratio was associated with CVD risk independent of hypertension, diabetes, obesity, and metabolic syndrome, and more risk attenuation was observed in healthy conditions. In addition, subjects with a higher LF/TF ratio and multiple CVD risk factors had a CVD risk comparable to those with a lower LF/TF ratio and few CVD risk factors. The association between LF/TF ratio and CVD risk remained significant after adjusting for other confounding factors.

Regional fat accumulation, rather than BMI, has a strong correlation with cardiometabolic disease incidence [10]. The role of each body fat depot to metabolic health seems to vary. Abdominal fat accumulation with visceral fat is adversely correlated with cardiometabolic disease [3, 23–25], accounted for disparate phenotype of subcutaneous and visceral adipocyte [26]. High visceral fat with low subcutaneous fat could be a marker for fat accumulation in vessel and liver, accelerating atherosclerosis and fatty liver [27, 28]. Furthermore, the role of subcutaneous fat seems to be diverse according to the body compartment. Deep subcutaneous adipose tissue was demonstrated to have similar phenotype of visceral fat, with increased insulin resistance and high CVD risk score, whereas superficial subcutaneous adipose tissue was the opposite [29]. Subcutaneous fat in the lower-body region has more potent properties of metabolism [30–32]. In healthy postmenopausal women, leg fat mass was a significant independent predictor of insulin sensitivity, reducing risk for hyperinsulinemia and insulin resistance, whereas trunk fat was associated with increased risk of those conditions [30]. In addition, this association was consistently maintained independent of body weight [31]. The results of our study support those of previous studies regarding the importance of body fat distribution as a predisposing risk factor for CVD and the extent to which LF/TF ratio is a better predictor compared to upper-body fat (ratio of arm fat to total fat) or trunk fat. Furthermore, we found that a high LF/TF ratio had stronger association with lower CVD risk when combined with sufficient leg muscle mass.

Previous studies using DXA provided limited results on the effect of regional adiposity distributions on CVD risk and were mainly confined to postmenopausal women. A study of postmenopausal women showed the association between regional fat and indirect CVD risk using hyperinsulinemia, and dyslipidemia [31]. Since then, a Danish group reports that peripheral fat mass correlated with aortic calcification in elderly females [33]. Both reports emphasized that localization of fat mass was more important than total body weight to atherosclerosis. The correlation between regional fat distribution on cardiometabolic risk factors, including blood pressure, HOMA-IR, high-sensitivity C-reactive protein, and dyslipidemia has been recently reported in a European study [30]. However, it too was limited to postmenopausal women and lacked of a comprehensive CVD risk assessment. To our knowledge, the present study is the first investigate on the impact of leg fat distribution on comprehensive CVD risk in a large general Korean population.

Although the biologic mechanism of each body fat depot has not been fully investigated, the different regional fat deposit seems to have different functional properties. Subcutaneous fat in the gluteofemoral region is considered a ‘metabolic sink’ due to its low rate of lipolysis, fatty acid uptake and blood flow [34]. In terms of adipose tissue lipolysis, the leg fat is the most sensitive adipose tissue to insulin [35]. These distinct features of lower-body fat prevent chronic exposure to high lipid concentration. In addition, adiponectin level and insulin sensitivity correlates with leg fat mass [32, 36]. Another study demonstrated the profound secretion of lipokine, palmitoleate from leg fat using venoarterial difference sampling [37]. A lower LF/TF ratio was associated with increased risk of each cardiometabolic risk factor component in our results. It was more closely related to the risk of obesity and diabetes than to hypertension and dyslipidemia, with the highest OR associated with metabolic syndrome, suggesting that a lower LF/TF ratio combined with another cardiometabolic risk factor component might synergistically increase its harmful effect. Furthermore, a lower LF/TF ratio was related to chronic kidney disease and even albuminuria risk in our results. Considering albuminuria as a marker of a premature stage of inflammation and CVD progression [38], a lower-body fat depot might be involved the in early development of CVD. As an interesting finding, individuals with multiple risk components and a higher LF/TF ratio had a CVD risk equivalent to those with a lower LF/TF ratio and few risk factors. The current study not only complemented the previous studies by showing a negative association of LF/TF ratio with CVD risk, but also revealed that higher LF/TF ratio is related with attenuated CVD risk even in high risk group. Moreover, the proportion at high risk was the highest in non-obese individuals in the lowest LF/TF ratio tertile, highlighting the importance of the LF/TF ratio in non-obese patients. These findings support previous studies suggesting the importance of the fat distribution effect to risk of CVD in people with normal BMI [39], and implying that regional fat can distinguish high CVD risk patients in a non-obese group [40, 41]. In non-obese group with diabetes, the correlation was not significant in our result might suggest the overwhelmed impact of diabetes on CVD.

To converse or attenuate the inappropriate impact of visceral fat, exercise should be recommended. 12-week standard aerobic exercise program effectively reduced visceral adipose tissue, and change in visceral fat area, but not subcutaneous fat was significantly associated with insulin resistance [42]. 1-year exercise and lifestyle modification program decreased visceral fat, but not subcutaneous fat, which resulted in enhancing adiponectin levels [43]. This impact of exercise was not limited in adult, but also was shown in obese adolescent individuals [44]. In addition, a multidisciplinary clinical trial including CT-scan, metabolomics, and transcriptomics suggested that promoting subcutaneous fat expansion could be a therapeutic option for reducing metabolic complications in obese individuals impending diabetes or cardiovascular diseases [45]. Restoring dysregulated inflammatory and oxidative phosphorylation pathways in the adipose tissue would be a promising target for the treatment of atherosclerosis and insulin resistance [46].

This study has some limitations. First, due to its cross-sectional design, we could not assert direct causality between LF/TF ratio and CVD risk. In addition, we applied indirect parameters to assess CVD risk. However, we used multiple surrogate CVD risk models with detailed statistical analyses to overcome this issue. Second, KHNASE did not collect each individual’s medication information, and the potential medication effect on CVD risk was not analyzed. Third, DXA could not exactly distinguish between subcutaneous fat and intramuscular fat.

Despite these limitations, our study has several strengths. First, it utilized data from a large, national, population-based study, guaranteeing statistical reliability without selection bias. We were able to divide sex-specific LF/TF ratio tertiles, whereas previous studies on lower-body fat were conducted mostly in women. Second, various equations and adjustment models were applied and they consistently showed similar results. Third, we limited the study population by excluding those with prior CVD history, to prevent reverse causality. The novelty of our results was that LT/TF ratio was superior to other regional fat depots as a predictive marker of high CVD risk. Furthermore, CVD risk lowering impact of LF/TF ratio was more prominent among individuals with high CVD risks.

The LF/TF ratio may distinguish individuals with a high CVD risk and provide reinforced risk modification to those subjects. In addition, our finding that a higher LF/TF ratio might have a beneficial effect on CVD risk, could be a clue for “obesity paradox” which refers the decreased risk of death in overweight individuals compared to those in normal weight. Although we only analyzed adult population in this study using DXA, technical assessment of body composition [47], and association between regional fat depots and cardiometabolic risk in obese adolescents needs to be further investigated. Prospective, well-designed, longitudinal studies with sufficient laboratory and cardiovascular imaging resources are warranted to identify the complex relationship between LF/TF ratio and CVD risk.

## Conclusions

This nationwide survey of a representative sample of Korean population demonstrated that LF/TF ratio was important for predicting higher CVD risk and, was associated with increased risks of CVD, independent for other cardiovascular factors.

## Additional files

**Additional file 1: Figure S1.** Proportion of individuals with CVD risk score tertiles by LF/TF ratio.

**Additional file 2: Figure S2.** Difference in ACC/AHA ASCVD risk according to LF/TF ratio tertiles. ACC/AHA ASCVD risk stratified by **A** obesity, and **B** age. Proportion of individuals with high ACC/AHA ASCVD risk (>10%) stratified by **c** obesity, and **d** age.

**Additional file 3: Figure S3.** Difference in Framingham CVD risk according to LF/TF ratio tertiles, subgroup analysis. Proportion of individuals with high CVD risk (>20%) stratified by **A** hypertension, **B** diabetes, **C** metabolic syndrome, and **D** insulin resistance (HOMA-IR). **E** Number of cardiovascular risk factors according to LF/TF tertiles. Risk factors are obesity, hypertension, diabetes, hyper LDL-cholesterolemia, and hypertriglyceridemia.

**Additional file 4: Figure S4.** Difference in Korean CHD risk according to LF/TF ratio tertiles, subgroup analysis. Proportion of individuals with high CHD risk (>5%) stratified by **A** hypertension, **B** diabetes, **C** metabolic syndrome, and **D** insulin resistance (HOMA-IR). **E** Number of cardiovascular risk factors according to LF/TF tertiles. Risk factors are obesity, hypertension, diabetes, hyper LDL-cholesterolemia, and hypertriglyceridemia.

## Abbreviations

ACC/AHA: American College of Cardiology/American Heart Association
ANOVA: analysis of variance
ASCVD: atherosclerotic cardiovasuclar disease
BMI: body mass index
CHD: coronary heart disease
CI: confidence interval
CVD: cardiovascular disease
DXA: dual-energy X-ray absorptiometry
eGFR: estimated glomerular filtration rate
HDL: high density lipoprotein
HOMA-IR: homeostasis model assessment of insulin resistance
hs-CRP: high-sensitivity C-reactive protein
KNHANES: Korea National Health and Nutrition Examination Surveys
LDL: low density lipoprotein
LF/TF ratio: leg fat to total fat ratio
OR: odds ratio
