# Supplementary figures and images for: Anatomic fat depots and cardiovascular risk: a focus on the leg fat using nationwide surveys (KNHANES 2008–2011)
Source: Cardiovasc Diabetol. 2017 Apr 26;16:54. doi: 10.1186/s12933-017-0536-4 (PMC5405479; doi:10.1186/s12933-017-0536-4)

A

Framingham CVD score, tertile

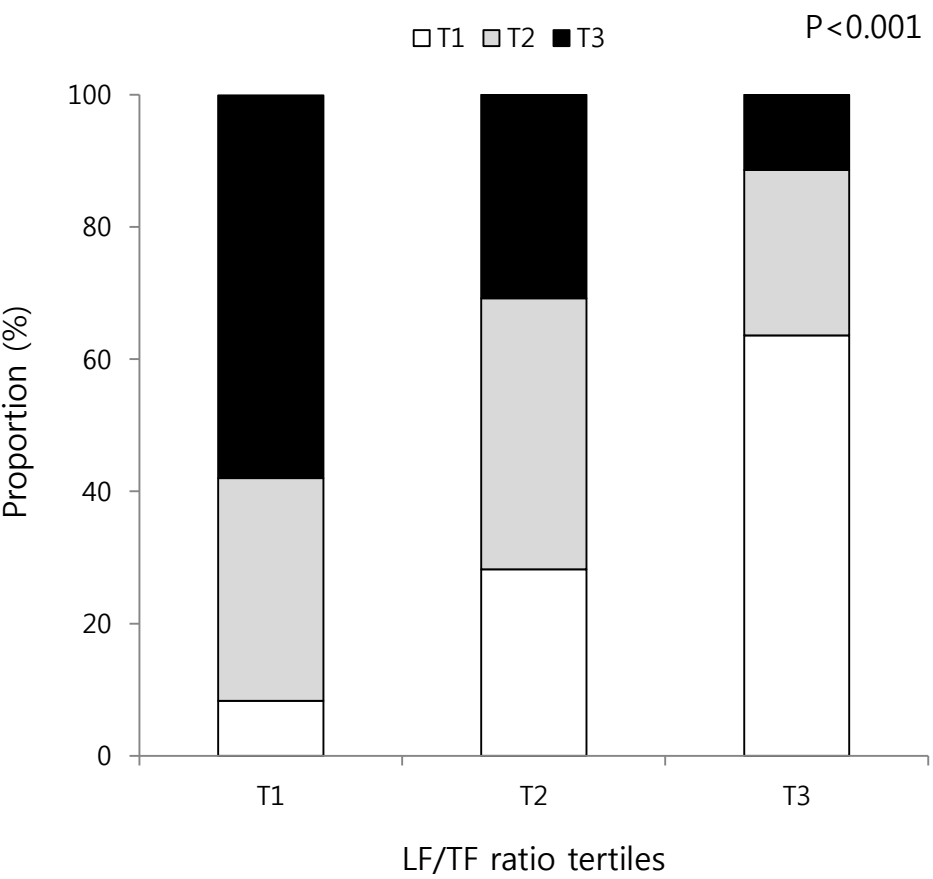

B

Korean CHD score, tertile

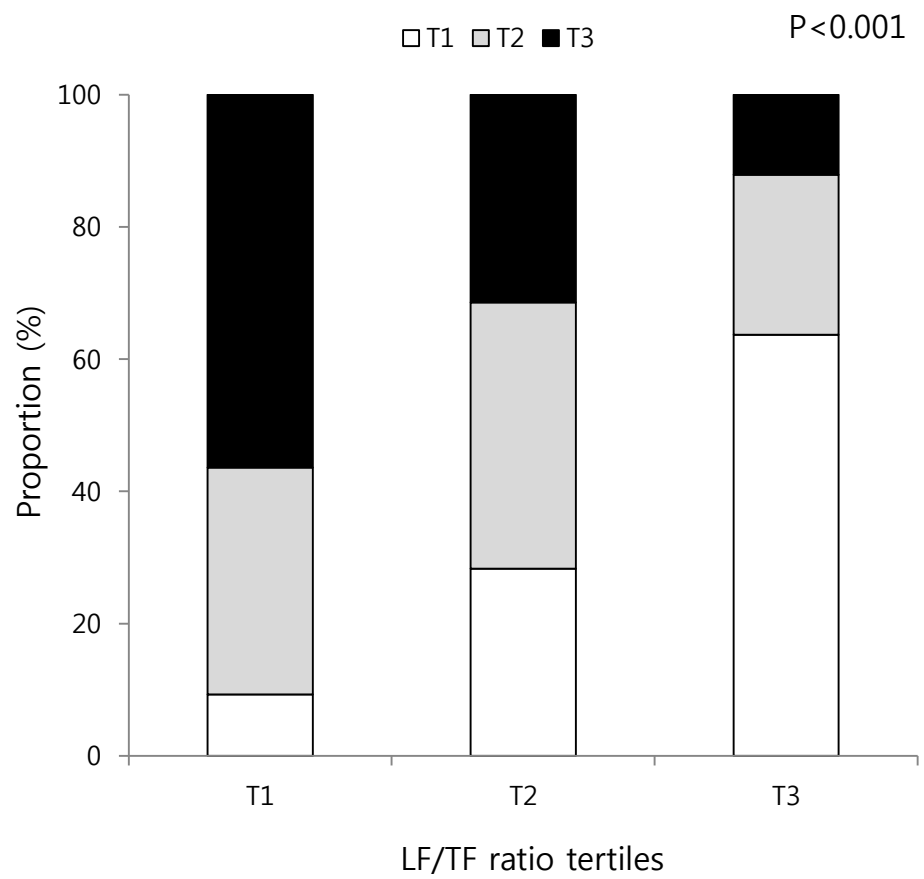

Supplement: Supplementary file 1 — Additional file 1: Figure S1. Proportion of individuals with CVD risk score tertiles by LF/TF ratio. [file 12933_2017_536_MOESM1_ESM.pdf]

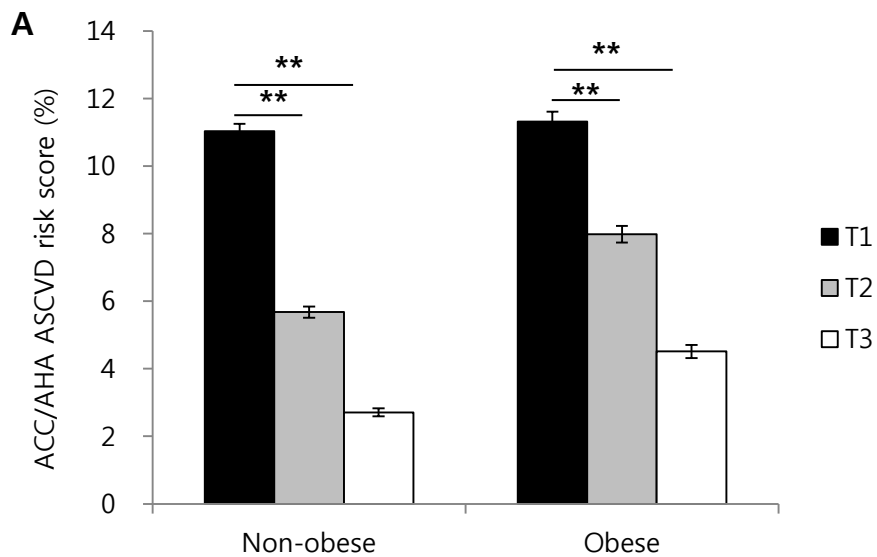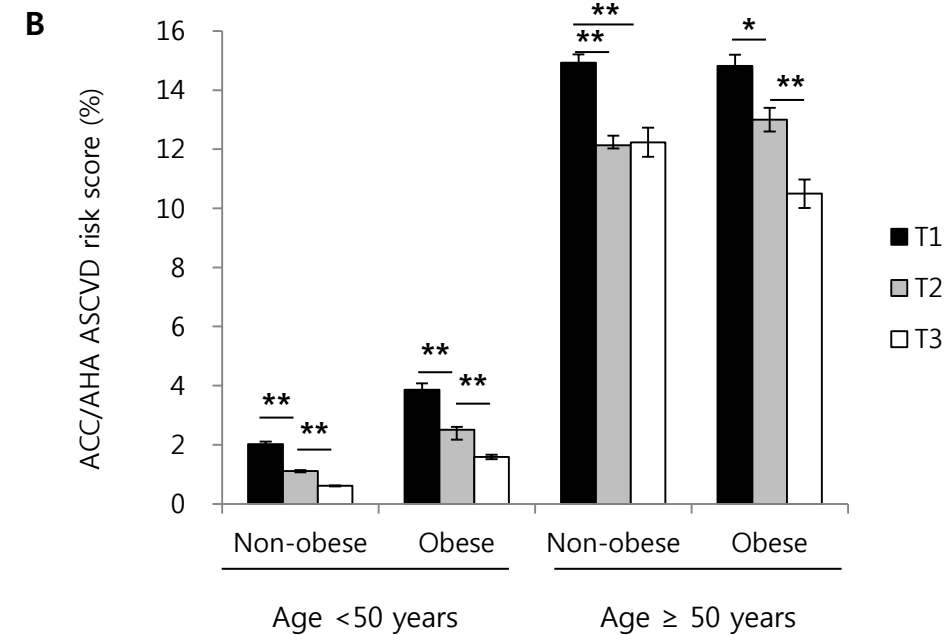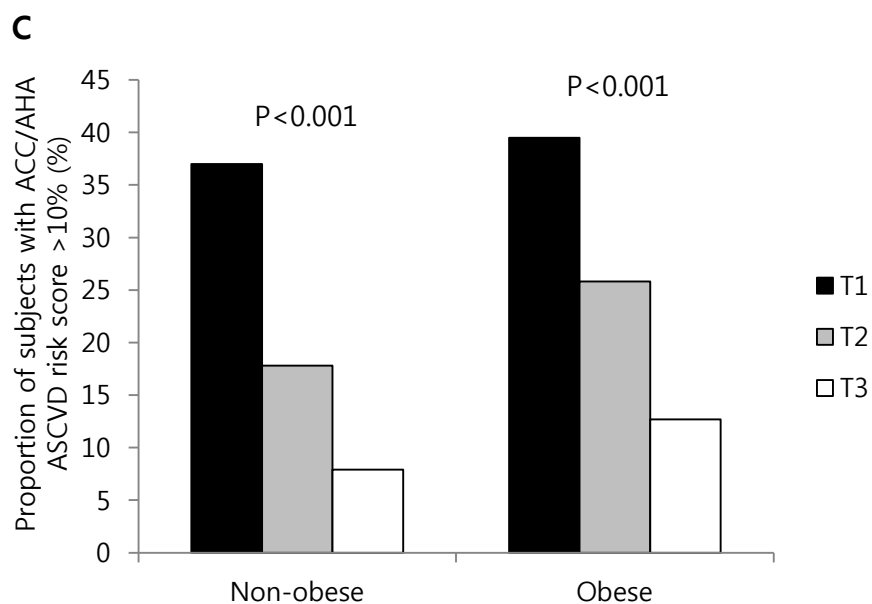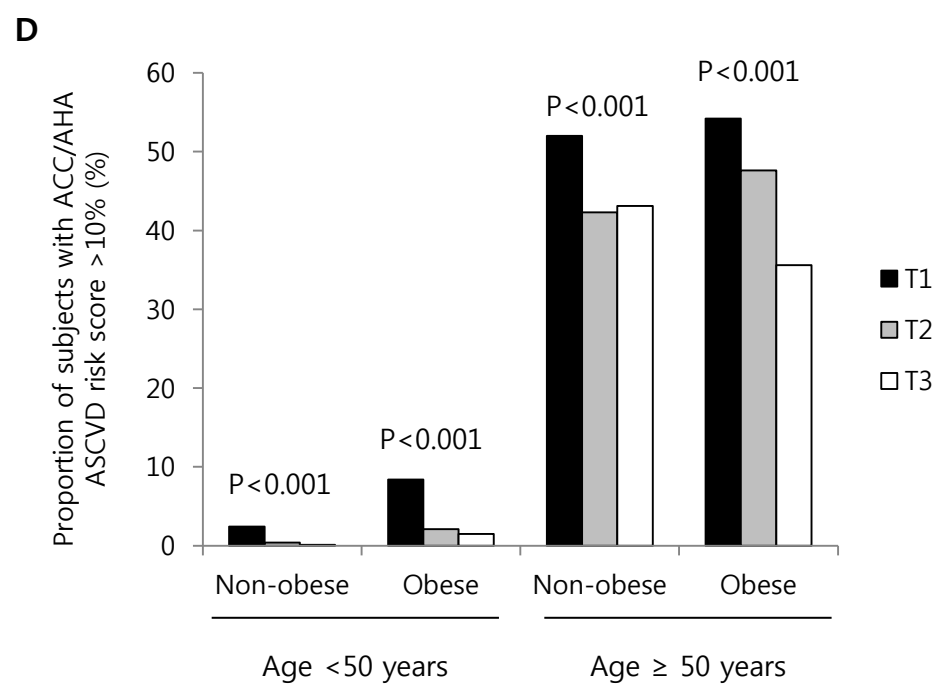

Supplement: Supplementary file 2 — Additional file 2: Figure S2. Difference in ACC/AHA ASCVD risk according to LF/TF ratio tertiles. ACC/AHA ASCVD risk stratified by A obesity, and B age. Proportion of individuals with high ACC/AHA ASCVD risk (>10%) stratified by c obesity, and d age. [file 12933_2017_536_MOESM2_ESM.pdf]

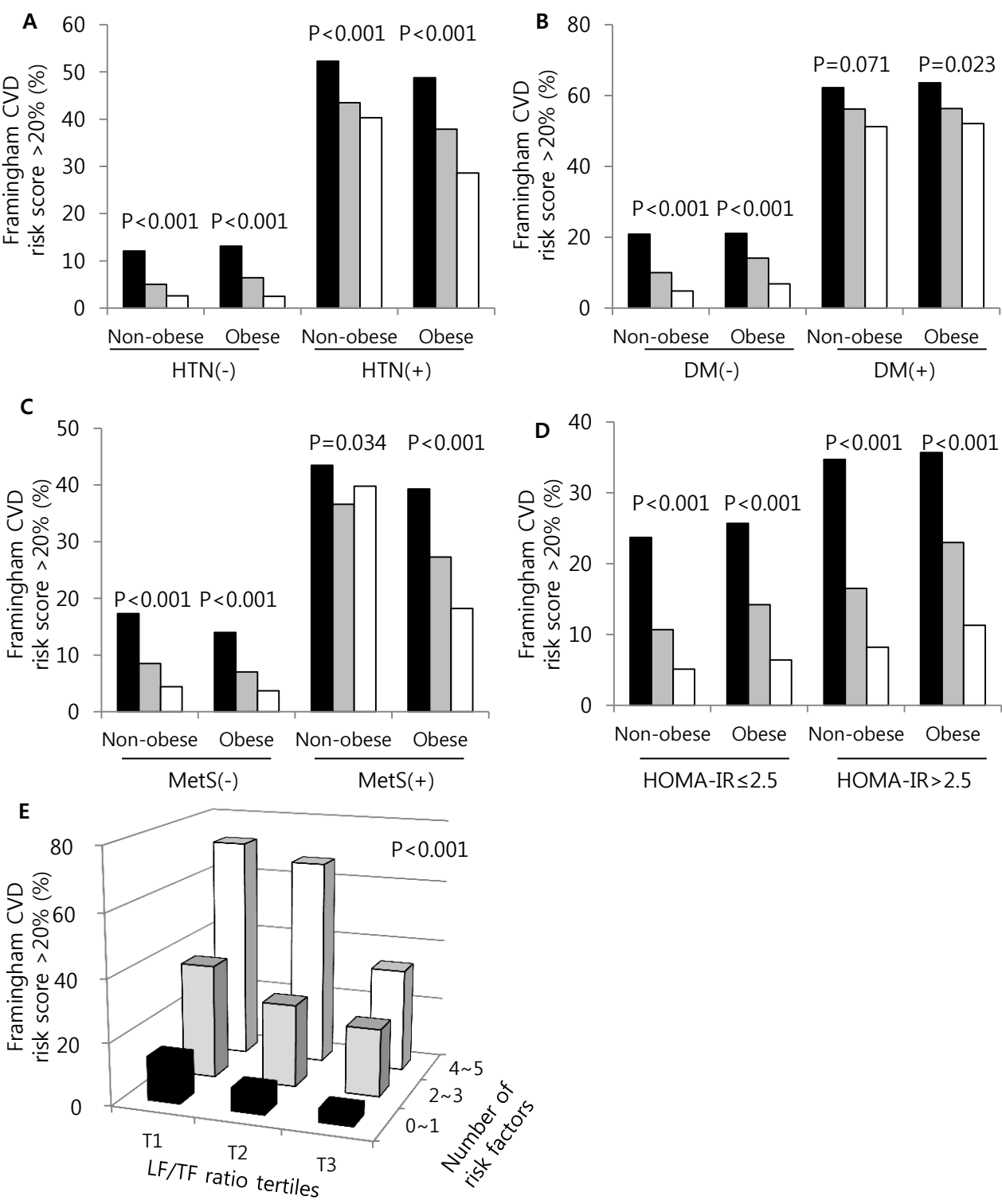

Supplement: Supplementary file 3 — Additional file 3: Figure S3. Difference in Framingham CVD risk according to LF/TF ratio tertiles, subgroup analysis. Proportion of individuals with high CVD risk (>20%) stratified by A hypertension, B diabetes, C metabolic syndrome, and D insulin resistance (HOMA-IR). E Number of cardiovascular risk factors according to LF/TF tertiles. Risk factors are obesity, hypertension, diabetes, hyper LDL-cholesterolemia, and hypertriglyceridemia. [file 12933_2017_536_MOESM3_ESM.pdf]

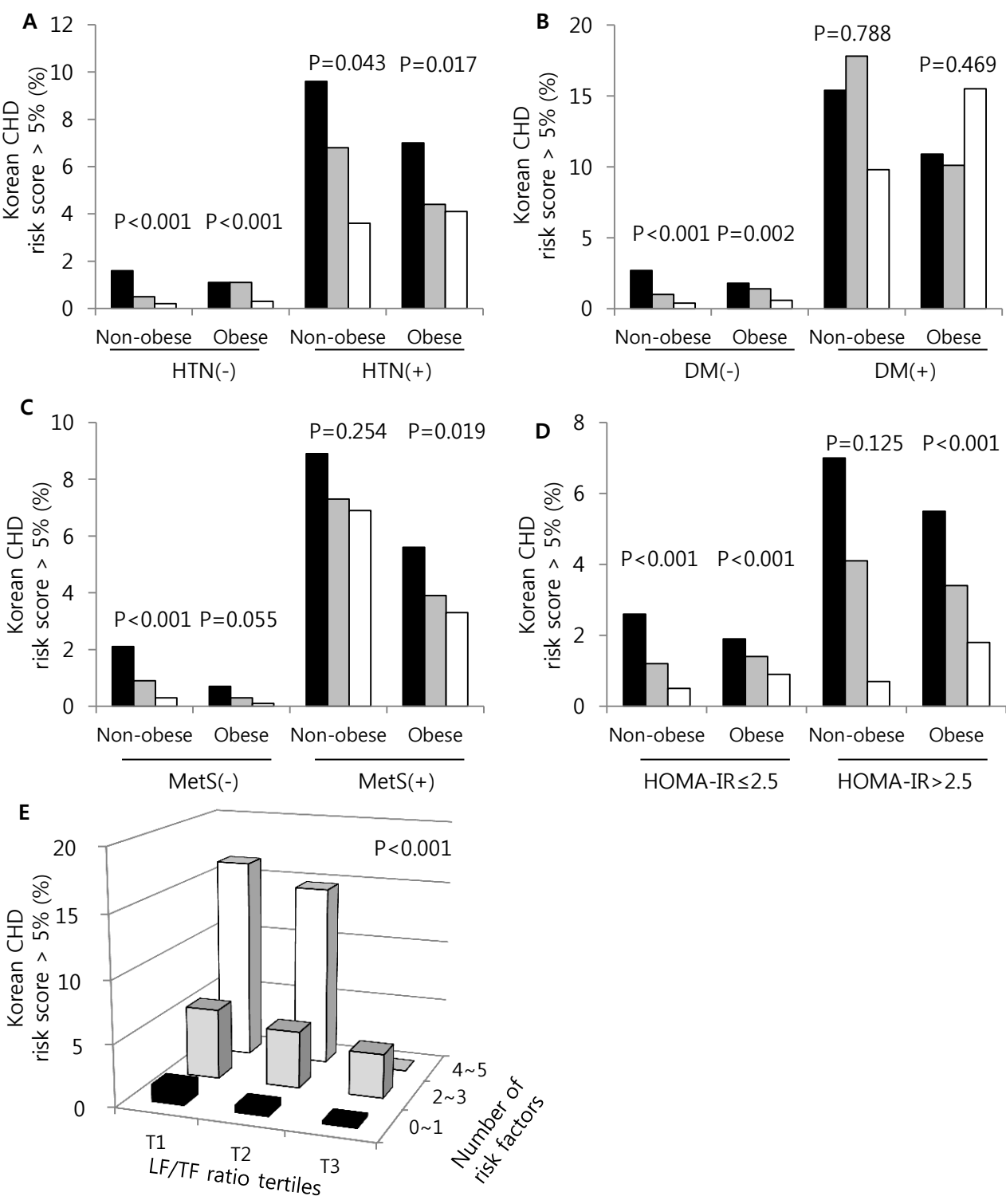

Supplement: Supplementary file 4 — Additional file 4: Figure S4. Difference in Korean CHD risk according to LF/TF ratio tertiles, subgroup analysis. Proportion of individuals with high CHD risk (>5%) stratified by A hypertension, B diabetes, C metabolic syndrome, and D insulin resistance (HOMA-IR). E Number of cardiovascular risk factors according to LF/TF tertiles. Risk factors are obesity, hypertension, diabetes, hyper LDL-cholesterolemia, and hypertriglyceridemia. [file 12933_2017_536_MOESM4_ESM.pdf]
